# Aggressive Primary Cutaneous Anaplastic T-Cell Lymphoma Successfully Treated with Autologous Stem Cell Transplant and Brentuximab Vedotin Consolidation: Case Report and Review of the Literature

**DOI:** 10.3390/hematolrep14020010

**Published:** 2022-03-23

**Authors:** Luca Guarnera, Federico Meconi, Marco Pocci, Fabiana Esposito, Manuela Rizzo, Vito Mario Rapisarda, Annagiulia Zizzari, Cosimo Di Raimondo, Livio Pupo, Lucia Anemona, Maria Cantonetti

**Affiliations:** Department of Biomedicine and Prevention, University Tor Vergata, 00133 Rome, Italy; federico.meconi89@gmail.com (F.M.); marco.med@hotmail.it (M.P.); fabiana.e91@gmail.com (F.E.); manuela.rizzo@ptvonline.it (M.R.); vito.rapisarda.89@gmail.com (V.M.R.); annadot86@hotmail.it (A.Z.); cosimodiraimondo@gmail.com (C.D.R.); livio.pupo@ptvonline.it (L.P.); anemona@uniroma2.it (L.A.); cantonetti@med.uniroma2.it (M.C.)

**Keywords:** cutaneous lymphoma, anaplastic T-cell lymphoma, autologous stem cell transplant, brentuximab vedotin

## Abstract

Primary cutaneous CD30+ lymphoproliferative disorders include primary cutaneous anaplastic large cell lymphoma (pcALCL) and lymphomatoid papulosis. The prognosis of the disease is usually excellent but, in a minority of cases, it presents with extracutaneous involvement and aggressive behavior. The case we present—relapsed after surgical excision, immunosuppressive therapy, and conventional chemotherapy—is the first one treated with Autologous Stem Cell transplant followed by Brentuximab Vedotin consolidation, a scheme already used for high risk Hodgkin Lymphoma.

## 1. Introduction

Primary cutaneous lymphomas (PCLs) are a heterogeneous group of lymphomas characterized by primary involvement of the skin. The most common subgroups are mycosis fungoides (MF) (75–80%) and the primary cutaneous CD30+ lymphoproliferative disorders (pcCD30+ LPDs, 20–25% of PCLs).

The group of pcCD30+ LPDs includes primary cutaneous anaplastic large cell lymphoma (pcALCL) and lymphomatoid papulosis (LyP) [1].

pcALCL occurs predominantly in males (M/F:3/1), and the common age of presentation is the sixth decade. Immunodeficiency seems to be a risk factor for this disease [2].

pcALCL clinically presents with solitary nodules or papules, sometimes ulcerated. In 20% of cases, there are multiple lesions. In 10%, there is extracutaneous involvement, usually with aggressive behavior. Patients with multifocal skin lesions are more likely to present extracutaneous involvement [3].

Mechanisms and molecular features that cause extracutaneous involvement and aggressive behavior are not yet clear.

Rare cases of ALK+ pcALCL seem to have a more aggressive course as well as tumors with re-arrangement of p53 and with CDKN2a-CDKN2B deletions [4,5,6].

The homing mechanism of cancer cells, likely lost in extracutaneous disease evolution, has been identified by some studies in the expression of the antigen HECA-452 (cutaneous lymphocyte associated antigen, CLA) although agreement on this point is not unanimous [7,8].

Diagnosis is based on histological examination of skin lesion biopsy. The histological framework can be very heterogeneous. Classically, it is possible to observe a dermal infiltration with large lymphocytes in which CD30 is expressed in more than 75% of cells (hallmark of disease), often with positivity of CD3 and CD4 and variable expression of CD5 and CD2. In about half of the cases, it is also possible to find cytotoxic markers (TIA1, perforin, and granzyme B) [3,9]. Many of the large cells present anaplastic nuclear features, with large and irregularly shaped hyperchromatic or vesicular nuclei with one or more nucleoli (similar to those seen in the Reed-Sternberg cell of Hodgkin Disease) and abundant slightly basophilic cytoplasm [10].

The recommended therapies are surgical excision and/or involved site radiotherapy (ISRT) or ISRT for solitary or grouped lesions. For multifocal lesions or pcALCL with regional nodes, systemic therapy is recommended. In the case of refractoriness or recurrence, treatment with the same regimen (unless refractory or intolerant) or an alternative regimen are both possible choices [11].

In the case of systemic ALCL, therapy and prognosis are variable depending on the expression of anaplastic lymphoma kinase (ALK): ALK+ ALCLs have a better prognosis and respond better to therapy. First-line treatment is polychemotherapy. In young patients, autologous stem cell transplantation is recommended as consolidation in ALK− ALCLs. In the case of relapse/refractoriness, salvage therapy involves the use of Brentuximab Vedotin (BV) if CD30 is expressed; otherwise, further chemotherapy is required [12,13].

pcALCL has an excellent prognosis, with a 10-year survival of 90% [3].

Negative prognosis factors are extracutaneous involvement; resistance to therapy; and presentation in specific areas, such as arms and legs. Age at diagnosis and extent of skin lesions seem not to affect the prognosis. There is no unanimous consensus on local lymph node involvement as a prognostic factor [3,14].

Skin relapse after treatment is a common condition (40–50%), with a discrete specific survival of 64% (reported from the Spanish group of Lymphoma). There is a lack of data available about the prognosis of relapsing extracutaneous diseases. Disease-related death occurs between 50 and 75% of cases, with a low complete response rate of 14% (reported from ALCANZA study) [3,14,15,16].

We herein report the case of a patient with aggressive pcALCL successfully treated with autologous stem cell transplant followed by BV consolidation.

## 2. Case Report

A 45-year-old man with a 2-year history of pcALCL was referred to our institution in February 2015 for enlargement of multiple left axillary lymph nodes (maximum diameter 4 cm).

**Figure 1:**
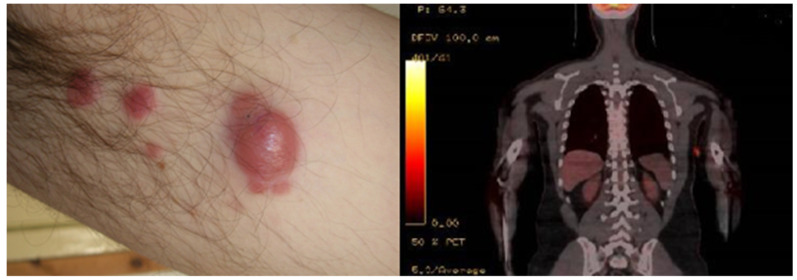
Lesion on the left armpit at onset of disease.

Two years before, he had developed multiple and isolated papular and nodular cutaneous lesions in the left armpit, the ipsilateral forearm, and the right calf. Lesional skin biopsy had showed a conspicuous infiltration in both deep and superficial dermis by immunophenotyping atypical limphocytes CD5+, CD20−, CD30+, PAX-5−, and ALK−. Final diagnosis was primary cutaneous CD30+ T-cell non Hodgkin Lymphoma (Figure 1).

Therefore, the patient was treated with excisional surgery, systemic steroid, and methotrexate, resulting in complete but short-term responses. In August 2014, the patient received local radiotherapy (RT) at the left forearm (Gy 4000), with disappearance of residual largest cutaneous lesion.

At the time of our observation, a whole-body positron emission tomography/computed (PET/TC) confirmed a marked ^18^F-Fluorodeoxyglucose (FDG) intake at the left axillary lymph nodes. A biopsy was performed on the largest lymph node. The histology showed a monomorphic cell population consisting of large blastic elements (CD20−, CD3+, CD4+, CD5+, CD30+, ALK1−, CD8−, c-Kit−, Granzyme B−, PAX5−, CD2+, TCRBetaF1−) with focal areas of necrosis. Final diagnosis was CD30+ Anaplastic T-cell non Hodgkin Lymphoma, demonstrating the pcATCL evolution in nodal disease. Bone marrow presented negative for disease involvement. Inflammatory markers—namely LDH, ferritin, fibrinogen, and β2 microglobulin—were within limits.

The patient then started a CHOEP polychemotherapy regimen. After the second of five cycles, the patient received one cycle of DHAP polichemotherapy (Cisplatin, dexamethasone, cytarabine) to recruit and collect autologous Hematopoietic Stem Cells (HSC). After documented Complete Remission (with CT scan and physical examination), the patient received a RT to the left armpit (3600 Gy).

**Figure 2:**
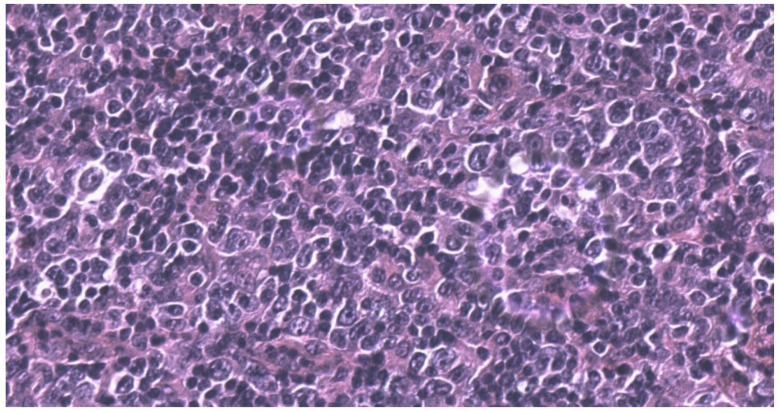
Inguinal lymph node biopsy. Lymph node architecture was completely effaced. Atypical lymphocytes were medium-large lymphoid cells with an irregular and atypical nuclei, with a prominent nucleoli and abundant cytoplasm. Hematoxylin and Eosin stain, 400×.

**Figure 3:**
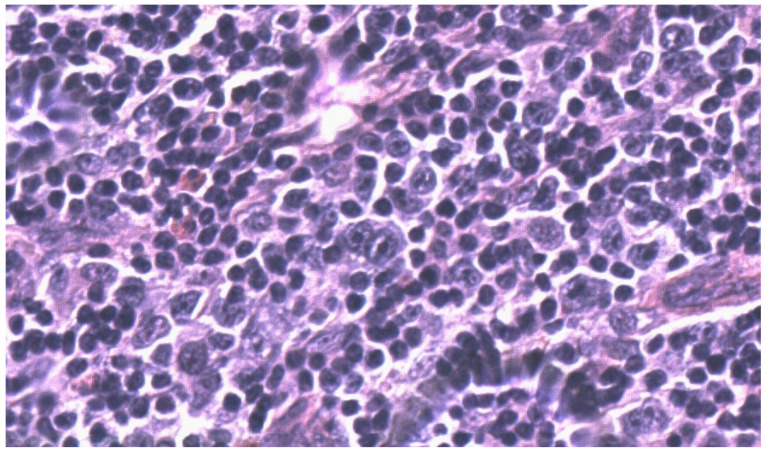
Inguinal lymph node biopsy. Scattered HRS-like cells were intermingled. Hematoxylin and Eosin stain, 600×.

Four months later, a PET/CT scan showed a significant FDG intake at the centimetric right inguinal round lymph node, and four new cutaneous lesions appeared at the left forearm. Histologic evaluation of the right inguinal lymph node showed the following: atypical lymphocytic population with medium-large-sized cells with oval or markedly irregular nucleus, clear chromatin, and evident nucleolus; a second population of large cellules with large irregular nucleus, some of them binucleated, and similar Hodgkin aspects was also detected. Immunohistochemical examination of the medium-large cells showed: PAX5+/−, CD3−, CD5−, CD2−, CD20−, MUM1+, CD30+, CD4+/−, CD8+, CD15−, LCA+, and BOB1−. Similar Hodgkin-Reed Sternberg cellspresented the following pattern: CD3−, CD5+, PAX5+, BOB1−, OCT2−, MUM1+, CD30+, CD2+, CD4+, LCA−, and CD15+/−. Morphological and immunohistochemical findings led to the diagnosis of “Non-Hodgkin’s anaplastic T-cell lymphoma with aberrant phenotype expressing aspects of the Hodgkin type” (Figure 2 and Figure 3).

In May 2016, the patient started treatment with BV, 1.8 mg/kg every 21 days for 6 cycles, obtaining a complete remission of disease (restaging with TC PET was negative).

In November 2016, the patient underwent autologous hematopoietic stem cell after a FEAM (Fotoemustine, etoposide, cytarabine, and melphalan) conditioning regimen.

Given the aggressiveness of the disease, we decided to administer 10 additional cycles of BV as consolidation therapy for a total of 16 cycles, with the last cycle in August 2017. No collateral effects were observed.

In October 2017, the complete restaging showed no residual disease, nor did the following clinical and imaging follow-up controls. Thirty months after last dose of BV, the patient is still in complete remission.

## 3. Discussion and Conclusions

Differential diagnosis between systemic ALCL and pcALCL can be difficult due to overlapping pathological features; in our case, the clinical history and presentation (primary skin involvement with spread to lymph nodes), together with the morphological and immunophenotypic picture (pathological lymphocytes in the dermis with typical CD4 expression), compatible with pcALCL, suggested this diagnosis [17].

Relapsing extracutaneous pcALCL is not a common disease, and there is lack of data and guidelines for it.

Indeed, NCCN guidelines refer to pcALCL with solitary/grouped lesions or multifocal lesions and to pcALCL with regional node, omitting pcALCL with organ involvement and not distinguishing forms with particularly aggressive behavior and with negative prognostic factors [11].

From the experience of Stanford University, collected in a monocentric study, it emerges that extracutaneous involvement (in addition to being a rare occurrence) is associated with a poor prognosis; three patients have been described, two of whom died and one of whom achieved complete remission thanks to systemic chemotherapy (5 CHOP-1CVP) [16].

In 2007, Isogai et al. described a case of a patient with an aggressive pcALCL with involvement of skin areas associated with poor prognosis (Femoral region, ulcerative lesion extending from the upper dermis to subcutaneous fat tissue) and nodal involvement without organ lesions, relapsed after chemotherapy with CHOP and successfully treated with a VNCOP-B regimen [18].

The Dutch Cutaneous Lymphoma group reported data on 2189 patients with primary and secondary CD30+ cutaneous lymphoproliferative diseases. Only 8 out of 79 pcALCL observed patients developed an extracutaneous disease. Their treatment was a CHOP regimen (6 patients) or autologous stem cell transplantation. After a median follow up of 61 months, 4 patients had died of lymphoma [3].

The treatment of these aggressive forms has been revolutionized by the introduction of BV, an anti-CD30 antibody conjugated to a synthetic antimicrotubule agent, monomethyl auristatin E. The common side effects are peripheral sensitive neuropathy, neutropenia, fever, diarrhea, vomiting, and fever. Neuropathy is one of the most disabling side effects and can lead to drug withdrawal [19].

In the ALCANZA study, 31 previously treated pcALCL patients were enrolled and randomly assigned (1:1) to receive intravenous BV 1.8 mg/kg once every 3 weeks, for up to 16 cycles, or the physician’s choice (oral methotrexate 5–50 mg once per week or oral bexarotene 300 mg/m^2^ once per day) for up to 48 weeks. Eleven of the enrolled patients presented extracutaneous pc-ALCL, four of which were treated with physician’s choice, achieving no objective response to the therapy. The remaining seven were allocated to the group of patients treated with BV, where four of them achieved an objective response lasting at least 4 months, and only one of them achieved complete remission of disease [15].

The efficacy of BV was also confirmed by André et al., who reported two cases of transformed Mycosis Fungoides successfully treated with BV as a bridge to Stem Cell Transplant and after transplant, for early recurrence [20].

Although several studies have been carried out demonstrating the effectiveness of BV or polichemotherapy in refractory/skin relapsing pcALCL, only a few case reports have been published about aggressive extracutaneous pcALCL therapies [15,21,22].

This case report represents the first one in the literature of management of the extracutaneous aggressive relapsing pcALCL treated with autologous stem cells transplantation and consolidation with BV, according to a scheme already authorized for high-risk Hodgkin Lymphoma, which has been proved to be an effective strategy in the most complicated cases [23].

## Data Availability

Not applicable.
